# Characteristic associated with alcohol drinking in early pregnancy: a cross sectional study

**DOI:** 10.1038/s41598-023-38055-3

**Published:** 2023-07-05

**Authors:** Kristina R. Saxov, Sofie Gry Pristed, Ulrik Schiøler Kesmodel

**Affiliations:** 1 Department of Clinical Medicine, Aalborg University, Aalborg, Denmark; 2 Department of Obstetrics and Gynaecology, Aalborg University Hospital, Reberbansgade 15, 9000 Aalborg, Denmark; 3 Programme of Biomedical Laboratory Science, University College of Northern Denmark, Hjørring, Denmark

**Keywords:** Health care, Risk factors

## Abstract

We aimed to identify characteristics associated with alcohol drinking before and during pregnancy to better target pregnancy guidance and public health campaigns. A cross sectional study including 1895 pregnant women interviewed at 16 weeks’ gestation. Information on characteristics and drinking habits before and during pregnancy was collected by in-person interview. Associations between characteristics and alcohol consumption were estimated by ordinal logistic regression models. Average alcohol intake *before* pregnancy was categorised; 0; > 0–3; > 3–6; > 6 drinks/week; and intake *during* pregnancy 0; < 1; 1–3; > 3 drinks/week; binge drinking 0; 1; 2; 3; ≥ 4 episodes. Characteristics for average alcohol intake before pregnancy were older age, odds ratio (OR) 3.99 (95% CI 2.77–5.74) when being 35 years or older. Schooling > 10 years, being primiparous and partner’s alcohol consumption were also significantly associated with average alcohol intake before pregnancy. Characteristics for average alcohol intake during pregnancy were age 25 to  < 35 years, OR 0.78 (CI 95% 0.61–0.98) and being single OR 1.52 (CI 95% 1.01–2.29). Characteristics for binge drinking during pregnancy were smoking OR 1.34 (CI 95% 1.06–1.69) when binge drinking was defined as ≥ 3 drinks/occasion and OR 1.49 (CI 95% 1.18–1.91) when defined as ≥ 5 drinks/ occasion. Other characterises found with a significant association were schooling > 10 years, being single, being primiparous and partner’s alcohol consumption. We identified characteristics that may be considered when counselling pregnant women or women planning to conceive. Public persuasive campaigns can be used to reach the general public, especially women of childbearing age, before they start planning to conceive, but also their partners, since women with partners consuming alcohol, did show to be more likely to consume alcohol during pregnancy.

## Introduction

In most countries, health authorities and health workers advise against alcohol consumption during pregnancy or when planning pregnancy^1,2^. The recommendations are based on the known risks of, e.g., miscarriage, malformations, low birth weight, and foetal alcohol syndrome associated with alcohol consumption^3,4^.

Most pregnant women tend to cease or reduce alcohol consumption when they recognise pregnancy; even so, studies have reported a prevalence of any alcohol drinking after pregnancy recognition reaching 60%^5,6^. Furthermore, up to 40% of pregnant women reported at least one episode of high alcohol intake, known as binge drinking^7,8^. Binge drinking most often occurs early in pregnancy and usually before the pregnancy is recognised. It has been found that 4% of pregnancy-aware women do not abstain from binge drinking^9^.

Several studies have assessed characteristics of women drinking during pregnancy. Even so, findings are inconsistent. Regarding maternal age some studies have shown advanced age in a reproductive perspective to be associated with alcohol consumption during pregnancy^10^, whereas other studies found no association ^11^. Smoking, education, and parity are also commonly examined characteristics; approximately 50% of studies showed that smoking was a characteristic for alcohol consumption, whereas the other 50% did not^12^. For education, some studies indicate a positive association with lower education, and other studies indicate a positive association with higher level of education^12^. Being married or having a partner has been shown to be associated with lower occurrence of prenatal alcohol consumption^13^, presumably because pregnancies are more often planned when in a stable relationship^14^.

It has been suggested that inconsistencies could be related to e.g. study design, geographical location, urban vs. non-urban settings, and questioning techniques^12^; but it is also possible that patterns of alcohol consumption may change over time^7,15^. While many studies have fair sample sizes, the actual participation rate is often unknown^14^ or rather low^11,13^ making room for selection bias of unknown magnitude and direction.

The aim of this study was to identify characteristics for average alcohol consumption before and during pregnancy and binge drinking during pregnancy based on two samples with high participation rates (> 90%), representing different geographical areas with different demographic characteristics, in order to assist health care providers to address the issue of alcohol in pregnancy in a relevant context. Further, aiming to elucidate the potential significance of cut-offs for the definition of binge drinking, two different definitions were used.

## Methods

### Settings

A total of 2053 Danish-speaking pregnant women who attended their first antenatal care visit with a midwife were invited to participate in the study. In Denmark, antenatal care visits with a general practitioner and a midwife are free of charge. Around 97% of pregnant women participate in the antenatal care programme^15^.

The study data consisted of a structured personal interview at gestational week 16 (median 16.8, interquartile range 13.9–21.7 weeks) at the first antenatal care visit with a midwife.

The structured interviews took place from 1 March to 31 August 2000 and were conducted by five research midwifes. All pregnant women (N = 2053) referred to the Midwife Centre in the university city of Aarhus (N = 1554) and in the smaller town of Frederica (N = 499) were invited to participate in the interview.

### Data collection

The interview mainly focused on alcohol drinking habits and knowledge about and attitudes towards alcohol intake during pregnancy. Questions were asked as regards average alcohol intake before and during pregnancy, different types of beer (non-alcoholic, light, normal, strong), wine, fortified wine, spirits, and alcopops (premixed alcohol and soda) Non-alcoholic beverages were coded as zero. The interview also included questions on the number of binge episodes within the first 20 weeks of pregnancy (but before the interview) and the gestational week when such binge episodes took place. Separate questions were asked about binge drinking defined as intake of ≥ 5 drinks on a single occasion^16^, which is the most used definition. Since it is anticipated that pregnant women and their foetuses are potentially vulnerable even to small amounts of alcohol, questions about intake of ≥ 3 drinks on a single occasion were also asked^17^. Furthermore, the interview included questions on other maternal characteristics such as weight before pregnancy and height, smoking habits, educational level and marital status and obstetric history including parity, previous miscarriages and stillbirths.

All interviews were performed either immediately after the first antenatal care visit or, if this was impossible, on a day of the woman’s own choice as soon as possible after the visit.

One drink was defined in accordance with Danish Health Authority as one standard drink equalling 12 g of pure alcohol, which is equivalent to one normal beer, one glass of wine, 8 cL of fortified wine or 4 cL of spirits.

Average alcohol intake *before* pregnancy was categorised into four groups (0; > 0–3; > 3–6; > 6 drinks/week) based on previous studies using > 6 drinks/week as the definition of frequent drinking^18^. Average alcohol intake *during* pregnancy was categorised into four groups for analyses (0; < 1; 1–3; > 3 drinks/week) based on studies reporting serious adverse events at levels of 4–5 drinks/week^19^. The partner’s average alcohol consumption was defined according to the guidelines and recommendations from the Danish Health Authority at the time the study took place (0; > 0–21, > 21 drinks/week). Consuming more than 21 drinks/week for men was considered heavy drinking^20^.

Gestational age at delivery, birth weight of the child, and paternal age were obtained from the Danish Medical Birth Registry^21^.

### Statistical analyses

Ordinal logistic regression was used for analysing the association between average alcohol consumption and lifestyle factors. The association between number of binge episodes (categorised as 0; 1; 2; 3; ≥ 4 episodes) with intake of either ≥ 3 or ≥ 5 drinks and lifestyle factors was also analysed from ordinal logistic regression.

We included the following potential characteristics in the analyses; maternal age (< 25 (reference), ≥ 25 to  < 35, ≥ 35 years), pre-pregnancy body mass index (BMI) (< 18.5 (reference), ≥ 18.5 to  < 25, ≥ 25 to  < 30, ≥ 30 to  < 35, ≥ 35), smoking (yes/no (reference)), school education (≤ 10 (reference), > 10 years), marital status (married (reference)/cohabiting or single), parity (primi-/multiparous (reference)) and partner’s number of drinks/week (0 (reference), > 0–21, > 21). All characteristics were simultaneously and mutually adjusted for all other characteristics, except paternal alcohol intake, which was analysed in a separate model excluding women with no partner.

Since no differences were observed in the associations between alcohol intake and the above characteristics between the two centres, only results using pooled data are presented.

Stata version 17 was used for data analysis (StataCorp 2021. Stata Statistical software: Release 17. College Station,TX: StataCorp LLC).

### Ethical approval

The study was approved by the Regional Ethics Committee (Den Videnskabsetiske Komité for Aarhus Amt, project no. 20000009) and the Danish Data Protection Agency (Datatilsynet, J.no. 2000-41-0259). All women gave written informed consent.

The study was performed in accordance with relevant guidelines and regulations.

## Results

A total of 2053 pregnant women were invited to participate of whom 1895 (92%) agreed to be interviewed.

The mean age of the women was 29.7 years at the time of the interview. The majority were married or co-habiting (95.4%), had more than 10 years of education (63.3%), and were non-smokers (69.3%). Half of the women were pregnant for the first time (Table 1).

**Table 1:** Characteristics of the interviewed women (N = 1895) Denmark, year 2000.

Characteristics	Total N (%)	Mean (SD)/Median (IQR)
Mean maternal age, years	1895 (100)	29.6 (4.4)
< 25	263 (13.9)	
25 to < 35	1408 (74.4)	
≥ 35	224 (11.7)	
Mean paternal age, years	1853 (98)	31.8 (5.2)
Pre-pregnancy body mass index (kg/m^2^)		
< 18.5	99 (5.2)	
≥ 18.5 to < 25	1337 (70.6)	
≥ 25 to < 30	326 (17.2)	
≥ 30 to < 35	98 (5.2)	
≥ 35	35 (1.8)	
Mean birthweight of child, grammes	1871 (99)	3554 (570.7)
Median gestational age at delivery	1877 (99)	40.4 [39.2; 41.4]
Smokers (before pregnancy)	583 (30.7)	
Smokers (during pregnancy)	329 (17.36)	
Median number of cigarettes/day before pregnancy (min, max)	533 (91.4)	13 (1, 40)
Median number of cigarettes/day during pregnancy (min, max)	312 (95)	8 (1, 25)
Years of education		
< 10 years	696 (36.7)	
Marital status		
Married or co-habiting	1808 (95.4)	
Parity		
Primiparous	960 (50.6)	
Partner’s average number of drinks/week		
0	92 (5.0)	
> 0–21 drinks/week	1731 (94.4)	
> 21 drinks week	10 (0.6)	

*SD* standard deviation, *IQR* interquartile range.

The differences in characteristics at the beginning of pregnancy between the two centers were seen in BMI, smoking, and education level. In Aarhus, the women had lower BMI (22 kg/m^2^), 38.4% smoked before pregnancy and 13% smoked during pregnancy, while women in Frederica had higher BMI (23 kg/m^2^), 37.6% smoked before pregnancy, and 29% during pregnancy. Regarding educational level, 71.1% of the women in Aarhus had more than 10 years schooling compared with 38.4% in Fredericia.

### Average alcohol consumption

All 1895 women were included in the analysis of average alcohol consumption before and during pregnancy. Of these, 1319 (70%) women consumed 0.5 drink per week or more during pregnancy. 1617 (85%) women reduced their intake after pregnancy recognition. For detailed information on pattern of alcohol consumption, Table 2. In total, 1830 (97%) gave information regarding their partner’s average alcohol consumption before pregnancy, while 65 women did not know about their partner’s drinking habits or did not have a partner at all.

**Table 2:** Numbers of participants in each category of alcohol consumption (N = 1895 for average alcohol consumption and N = 1836 for binge drinking) Denmark, year 2000.

Average alcohol consumption before pregnancy				
Drinks/week	0	> 0–3	> 3–6	> 6
Participants	120	762	589	424

Average alcohol consumption during pregnancy				
Drinks/week	0	< 1	1–3	> 3
Participants	576	451	782	86

Binge drinking during pregnancy*				
	≥ 3	≥ 5		
Numbers of episodes				
0	599	923		
1	500	527		
2	312	221		
3	160	85		
≥ 4	265	80		

Gestational week at binge drinking**				
1	308	184		
2	401	240		
3	527	336		
4	475	308		
5	336	206		
6–8	311	153		
≥ 9	212	62		

*Binge-drinking defined as intake of respectively ≥ 3 and ≥ 5 drinks on a single occasion.

**Each woman may contribute binge drinking during more than one week.

### Characteristics of women for alcohol consumption before pregnancy

Characteristics associated with alcohol consumption before pregnancy were age 25- < 35 (OR 2.33 (95% CI: 1.77–3.06)) or ≥ 35 years (OR 3.99 (95% CI: 2.77–5.74)) compared to women < 25 years. Smoking before pregnancy was also found to be characteristic for alcohol consumption before pregnancy (OR 1.52 (95% CI: 1.25–1.84)). So were school education > 10 years [OR 2.57 (95% CI: 2.12–3.11), being primiparous (OR 2.18 (95% CI: 1.82–2.59)] and partner’s average alcohol consumption [OR 15.29 (95% CI: 8.65–27.03)], Table 3.

**Table 3:** Association between average alcohol intake before and during pregnancy, lifestyle and socio-demographic characteristics.

	Before pregnancy			During pregnancy		
	N	OR	95% CI	N	OR	95% CI
Maternal age (years)	1895			1895		
< 25 (ref)	263	1.00			1.00	
25 to < 35	1,409	2.33	1.77–3.06		0.78	0.61–0.98
≥ 35	223	3.99	2.77–5.74		0.85	0.60–1.19
Prepregnancy BMI**						
< 18.5 (ref)	99	1.00			1.00	
≥ 18.5 to < 25	1,337	1.42	0.95–2.11		1.11	0.77–1.62
≥ 25 to < 30	326	1.05	0.68–1.63		1.11	0.74–1.67
≥ 30 to < 35	98	0.66	0.39–1.14		1.00	0.61–1.66
≥ 35	35	0.77	0.37–1.64		0.69	0.34–1.40
Smoking before pregnancy						
Non-smokers (ref)	1312	1.00				
Smokers	583	1.52	1.25–1.84			
Smoking during pregnancy						
Non-smokers (ref)				1566	1.00	
Smokers				329	0.95	0.75–1.19
Years of education						
≤ 10 (ref)	696	1.00			1.00	
> 10	1199	2.57	2.12–3.11		0.88	0.73–1.05
Marital status						
Married/cohabiting (ref)	1808	1.00			1.00	
Single	87	1.36	0.89–2.09		1.52	1.01–2.29
Parity						
Multiparous (ref)	935	1.00			1.00	
Primiparous	960	2.18	1.82–2.59		1.02	0.85–1.21
Partner’s average number of drinks/week***	1830			1833		
0 (ref)	62	1.00		92	1.00	
> 0–21	1732	15.29	8.65–27.03	1731	1.12	0.79–1.59
> 21	36	88.04	35.85–216.23	10	0.57	0.14–2.19

The associations are presented as adjusted odds ratios (OR)* from ordinal logistics regression models^†,‡^ (N = 1895) Aarhus and Frederica, Denmark, year 2000.

*All ORs mutually adjusted for the remaining factors in the table. Smoking before and during pregnancy not in the same model.

**Body mass index (kg/m^2^).

*** Partners consumption of alcohol. N ~ missing and females without a partner excluded.

^†^Number of drinks/week before pregnancy categorised into 0, > 0–3, > 3–6, > 6 drinks/week.

^‡^Number of drinks/week during pregnancy categorised into 0, > 0- < 1, 1–3, > 3 drinks/week.

### Characteristics for alcohol consumption during pregnancy

Women aged 25 to  < 35 years had a statistically significantly lower occurrence of drinking during pregnancy [OR 0.78 (95% CI: 0.61–0.98)] than women < 25 years. Being single was also found to be significantly associated with the occurrence of drinking during pregnancy [OR 1.52 (95% CI: 1.01–2.29)], Table 3.

### Binge drinking

Of the 1895 women, 59 did not answer the questions on binge drinking episodes. Consequently, 1,836 women were included in the analyses of binge drinking. In total, 1237 (67%) women reported binge drinking defined as ≥ 3 drinks on one or more occasion during pregnancy. 913 (50%) women reported binge drinking defined as ≥ 5 drinks on one or more occasion. Distribution of binge episodes are described in Table 2.

The 59 non-responders did not differ from the responders in terms of socio demographic factors or average alcohol consumption, but a significant difference in smoking during pregnancy was observed, showing higher smoking rates in the non-responder group (17 (28.8%) versus 312 (16.9%)).

Characteristics consistently associated with binge drinking irrespective of the definition used (≥ 3 or ≥ 5 drinks on a single occasion) were smoking during pregnancy, school education > 10 years, being single and primiparous, Table 4. Furthermore, the partner’s average alcohol consumption was also strongly associated with binge drinking ≥ 3 or ≥ 5 drinks on one occasion, Table 4.

**Table 4:** Association between the number of binge drinking episodes and lifestyle and socio-demographic factors.

		≥ 3 drinks		≥ 5 drinks	
	N = 1836	OR	95%CI	OR	95% CI
Maternal age (years)					
< 25 (ref)	256	1.00		1.00	
25 to < 35	1371	1.71	1.33–2.21	1.27	0.97–1.66
≥ 35	209	2.03	1.42–2.92	1.11	0.76–1.62
Prepregnancy BMI^†^					
< 18 (ref)	98	1.00		1.00	
≥ 18 to < 25	1294	0.89	0.61–1.33	0.77	0.51–1.15
≥ 25 to < 30	317	0.75	0.49–1.15	0.73	0.47–1.13
≥ 30 to < 35	92	0.50	0.29–0.86	0.68	0.39–1.18
≥ 35	35	0.58	0.29–1.17	0.77	0.37–1.57
Smoking during pregnancy					
Non-smokers (ref)	1524	1.00		1.00	
Smokers	312	1.34	1.06–1.69	1.49	1.18–1.91
Years of education					
< 10 (ref)	670	1.00		1.00	
> 10	1166	1.54	1.28–1.86	1.35	1.12–1.65
Marital status					
Married/cohabiting (ref)	1753	1.00		1.00	
Single	83	1.64	1.09–2.46	1.89	1.24–2.91
Parity					
Multipara (ref)	903	1.00		1.00	
Primipara	933	1.91	1.60–2.38	1.98	1.64–2.38
Partner’s average number of drinks/week**	1776				
0	88	1.00			
> 0–21	1678	3.70	2.35–5.84	3.37	2.01–5.65
> 21	10	11.06	3.21–38.08	4.77	1.23–18.08

The associations are presented as adjusted odds ratios (OR)* from ordinal regression models, (N = 1836). Aarhus and Frederica, Denmark, year 2000.

Binge-drinking defined as intake of respectively ≥ 3 and ≥ 5 drinks on a single occasion.

*All ORs are mutually adjusted for the remaining factors in the table.

**Based on women with a partner only (N = 1776).

^†^Body mass index (kg/m^2^).

If the definition of binge drinking ≥ 3 drinks on one occasion was used, we observed an association with higher age, whereas BMI between 30 and  < 35 was associated with lower occurrence of binge drinking (OR 0.50 (95% CI: 0.29–0.86)) than a BMI < 18.

## Discussion

The characteristics of women significantly associated with alcohol drinking before their pregnancy included age > 25 years, smoking, schooling > 10 years, being primiparous and partner’s average alcohol intake. The characteristics for women drinking during pregnancy were age < 25 years and being single. The lack of association observed between smoking and drinking during pregnancy aligns with the inconsistency found in earlier studies^12^.

In this study, we also found a statistically significant association in the occurrence of binge drinking among primiparous women. This association is well documented in earlier studies^9,22^. Episodes of binge drinking and alcohol consumption in general predominantly occur before recognition of pregnancy^9,23^.

Furthermore, our results on binge drinking align with earlier studies^9,10^ regarding smoking, marital status, maternal age, and educational level. These sociodemographic characteristics were all statistically significant, and they demonstrate that single, smoking, first-time pregnant women, higher aged and with longer education are more likely to binge drink than abstainers during pregnancy. Single women tend to live a more outgoing social life, consuming more alcohol than females in a relationship. It is well documented in earlier studies that binge drinking is associated with higher age and socioeconomic status, including longer education^9^. If a woman who has been living an outgoing life becomes pregnant, it is likely she will continue this lifestyle the way she did before she became pregnant, and continue to binge drink. Also, the risk of an unplanned pregnancy is presumable higher in this group, and smoking should be addressed before pregnancy when possible rather than being screened for at the first prenatal visit.

While we found no statistically significant association between BMI and binge drinking defined as ≥ 5 more drinks per occasion, there was a significantly lower occurrence of binge drinking defined as ≥ 3 drinks per occasion for women in the BMI category ≥ 30 to  < 35 kg/m^2^ compared to women with lowest BMI. The association for the BMI category ≥ 35 kg/m^2^ was of a comparable magnitude, albeit not statistically significant.

This could be explained by alcohol’s calorie density^24^. This group of women is presumably concerned about weight gain, and focused on not drinking avoidable calories, hence avoiding binge drinking.

In most countries, pregnant women are offered prenatal visits providing advice on alcohol abstinence, smoking cessation, vitamin supplementation, etc., which take place at the general practitioner or with a midwife around gestational week 6–10^25,26^. By that time, prenatal alcohol exposure has already taken place in at least 50% of the cases^8,27^. This leaves a gap in the timeline between alcohol exposure and the prenatal visits as organised in the present healthcare system. Given that it is a shared goal in healthcare and a societal duty to provide health information at the right time, we argue that our results raise the question whether pre-conceptional guidance on alcohol use should be introduced.

The timing of pre-conceptional consultation is a challenge since not all pregnancies are planned; even so, public information campaigns on the detrimental effects of alcohol consumption in early pregnancy and a general offer of pre-conceptional consultation at a young age may partly address this challenge^28^.

Even so, in many countries, pre-conceptional guidance is a targeted option reserved for women or men with known predictors, such as chronic diseases, mental illness or substance abuse^25,28^, often leaving women with no such predictors responsible for seeking relevant pre-conceptional guidance themselves in order to prevent poor perinatal outcomes.

Bearing in mind that a first-time pregnancy involves a higher occurrence of binge drinking, it seems that pre-conceptional guidance could benefit from being integrated into information campaigns targeting populations during early reproductive age. Early guidance would also provide information to younger aged women at risk of drinking during pregnancy. Guidance could be provided though public campaigns and more targeted education in high school and other educational institutions. With public campaigns, older, more well-educated women with higher tendency towards binge drinking would also be enlightened, and hence the guidance would benefit all women, across all age grouped.

During prenatal and pre-conceptional visits, it is important to clarify the partner’s alcohol consumption. This study reveals an important occurrence of maternal binge drinking while pregnant if the partner drinks alcohol. The result was both statistically significant and also of a substantial clinical relevance whether binge drinking was defined as ≥ 3 or ≥ 5 drinks on a single occasion. The strongest association was found if the partner drank ≥ 21 drinks peer week.

### Strengths

No valid biomarkers are available for measuring alcohol consumption in pregnancy; hence, self-reports are necessary. There is an ongoing debate regarding the merits and drawbacks of using self-administrated questionnaires or personal interviews to determine average alcohol intake, and the two methods seem to be equally good^29,30^; however, for binge drinking, personal interviews are suggested to be superior owing to higher response rates^27^.

Previous studies have shown that information on binge drinking becomes less valid when recall time is longer^31^. A validation study using the exact same data from Aarhus reported that many women consulted their personal diaries without being prompted to check the timing of binge episodes. Only very few women appeared uncertain of the accuracy of the information provided^27^. This could be due to the fact that all women in this study were interviewed in the first half of pregnancy, and it substantiates the reliability of their answers and reduces the potential problem with recall bias. Even if the problem with of recall bias is limited, it should still be considered.

### Limitations

Even though attempts have been made to reduce the risk of underestimation of alcohol consumption, a risk of underreporting introduced by the social norms still exists. Fifty-nine women did not respond to the questionnaire regarding binge drinking, which could possibly lead to underestimation. However, since no significant difference in average alcohol consumption was found between the responders and non-responders with respect to binge drinking, it is unlikely that selection bias is an issue. Nohr and colleagues have previously described that selection bias in relation to a frequent outcome has very little influence on the association in cohort studies; even if this is a cross-sectional study we believe that the same line off thought would be applicable, since alcohol exposure is frequent, and thus this study is unlikely to be influenced by selection bias to any relevant degree^32^.

Another limitation is the information on the partner’s drinking habits, which was estimated by the women in the present study, and for the highest intake group (> 21 drinks per week) there were relatively few male partners. Even so, the results indicated a strong association with the female partner’s average alcohol consumption before pregnancy and with binge drinking, although the confidence intervals indicate little precision. Hence, if the partner drinks (more than recommended), it is very likely that the woman has a high intake of alcohol. However, the public health and clinical importance of the estimates for all characteristics should be interpreted in the light of the confidence intervals, as some seemingly high estimates may in fact be of little real relevance, e.g. when the lower boundary is very close to one.

Regarding the statistical analysis, we did adjust for predefined characteristics, but we did not adjust for multiple comparisons. It is widely discussed if e.g., Bonferroni adjustment should be performed or not. While lack of adjustment might inflate the risk of type I error, adjustment could potentially increase the risk of type II error, so that differences in the results would be non-significant, and loss of important knowledge could occur^33^.

In this study, no attempts were made to clarify if the pregnancy was planned; hence, a high occurrence of binge drinking could be related to unplanned pregnancies. The unknown occurrence of unplanned pregnancy could also lead to residual confounding.

Furthermore, due to the cross-sectional design we were unable to assess the direction of any association and make causal inferences.

### Generalizability

While the two samples were not probability samples from the entire population they represented two different geographical areas, a large university city and a smaller regional town with different socio-demographic characteristics. While it is not possible to compare the distribution of all our data with the entire population of the time, available data suggest comparability with the distribution of maternal and paternal age, maternal education^34^ and birth outcomes^35^ at the time of data collection. Even so, even if the risk of selection bias and information bias is likely to be limited, as suggested above, some degree of bias may still exist, and this may influence the external validity.

## Conclusion

Women who are well educated, smokers, and with a partner consuming any amount of alcohol need to be identified to lower the prevalence of prenatal alcohol exposure. In this study the majority of women did cease alcohol consumption after pregnancy recognition. However, as binge drinking most often occurs before recognition of pregnancy, pre-conceptional guidance is needed in addition to prenatal care to reduce the occurrence of binge drinking. As regards women in a relationship, knowing the partner’s alcohol consumption is valuable to doctors and midwives, since the partner’s alcohol consumption is highly associated with the occurrence of binge drinking during pregnancy.

Future studies should assess if pre-conceptional guidance in relation to binge drinking may in fact reduce the occurrence of binge drinking before recognition of pregnancy.

## Data Availability

Access to data may be negotiated with the authors provided all relevant legal permissions can be obtained. The dataset is available from the corresponding author on reasonable request, provided relevant legal permissions for storing and handling data according to Danish legislation has been obtained.
